# Efficient Photoelectrochemical Reduction of CO_2_ in Seawater with Cheap and Abundant Cu_2_O/Al_2_O_3_/TiO_2_ Electrode

**DOI:** 10.3390/ma18030620

**Published:** 2025-01-29

**Authors:** Aleksandra Parzuch, Katarzyna Kuder, Kostiantyn Nikiforow, Piotr Wróbel, Grzegorz Kaproń, Krzysztof Bieńkowski, Renata Solarska

**Affiliations:** 1 Laboratory of Molecular Research for Solar Energy Innovations, Centre of New Technologies University of Warsaw, Banacha 2c, 02-097 Warsaw, Poland; a.parzuch@cent.uw.edu.pl (A.P.); k.kuder@cent.uw.edu.pl (K.K.); k.bienkowski@cent.uw.edu.pl (K.B.); 2 Institute of Physical Chemistry, Polish Academy of Science, Kasprzaka 44/52, 01-224 Warsaw, Poland; knikiforow@ichf.edu.pl; 3 Faculty of Physics, University of Warsaw, 02-093 Warsaw, Poland; piotr.wrobel@fuw.edu.pl; 4 Faculty of Geology, University of Warsaw, ul. Żwirki i Wigury 93, 02-089 Warsaw, Poland; gkapron@uw.edu.pl

**Keywords:** carbon dioxide reduction, copper oxide (I), aluminum oxide

## Abstract

The photoelectrochemical (PEC) reduction of carbon dioxide to environmentally friendly fuels is a promising strategy to address the challenge of clean energy demand. Semiconductor photocathodes such as Cu_2_O enable the reduction of carbon dioxide, but their main drawback is their instability and susceptibility to photocorrosion. In this work, Al_2_O_3_ and TiO_2_ were utilized to enhance stability, photoelectrochemical activity, and charge transport facilitation, resulting in a 2.8-fold increase in generated photocurrent density (1.4 mA/cm^2^ at −0.2 V vs. RHE). The experiments were conducted in a 0.5 M NaCl solution, simulating seawater conditions, to evaluate the performance and stability of the system in an environment closer to real-world applications

## 1. Introduction

Growing global energy demands have intensified carbon dioxide (CO_2_) emissions, the primary contributor to the greenhouse effect. Alongside this, rising environmental awareness has driven substantial interest in renewable solutions for CO_2_ capture and conversion, especially in alternative methods using solar-driven CO_2_ conversion to environmentally friendly fuels [1]. Closing the carbon cycle in nature is a huge challenge, and using renewable energy for this purpose is the most beneficial challenge. Semiconductors are promising materials for the conversion of solar energy, one of which is well known to be copper (I) oxide (Cu_2_O) [2]. Cu_2_O is a cheap, non-toxic, readily available p-type semiconductor with a narrow band gap of ~2.17 eV, absorbing visible light [3,4,5]. The convenient location of the valence and conduction bands allows both CO_2_ and water to be reduced [6]. The main disadvantages of Cu_2_O are its low stability in aqueous solutions, susceptibility to photocorrosion, and the ability to oxidize and reduce itself [7]. This semiconductor also shows high selectivity for the reduction of CO_2_ to methanol [8,9,10,11]. To overcome the problems associated with Cu_2_O, i.e., low stability and photocorrosion, as already mentioned, many attempts have been made to stabilize and extend the life of Cu_2_O, as well as to diversify the CO_2_ reduction products. Commonly, various types of protective coatings, such as aminopolysiloxane, are employed to enhance the stability of Cu_2_O. Z. Yu et al. used polysiloxane-containing amines to form hydrophobic AF-Psi chains on the surface of Cu_2_O, which effectively mitigates photocorrosion caused by the presence of water in the electrolyte. The presence of a nitrogen atom makes it possible to break the symmetry of the CO_2_ molecule and obtain 61% of the Faradaic efficiency to formate [12]. Also, n-type oxide semiconductor outer layers are commonly used such as TiO_2_, ZnO, SnO_2_, and other interlayer oxides, namely NiO, and Al_2_O_3_ [13,14,15,16]. W.Z. Tawfik et al. modified Cu_2_O by ZnO with a water splitting reaction. Cu_2_O/ZnO creates heterojunction photogenerated electrons in the conduction band of Cu_2_O flow down to a lower-lying conduction band of ZnO and next to the electrolyte, where they can reduce H_2_O. Holes from the valance band of ZnO are moving into VB of Cu_2_O and to the electrode substrate. This process inhibits the recombination of photogenerated hole–electron pairs [13]. TiO_2_ is highly favored for its widespread use thanks to its porous and high surface area, which promotes the favorable adsorption of CO_2_ on the electrode surface and protects Cu_2_O against photocorrosion. The combination of p-type Cu_2_O and n-type TiO_2_ leads to the formation of a heterojunction, enabling the reduction in recombination in photogenerated charges [14]. SnO_2_ also works as a protective layer from photocorrosion, improving the stability creating p-n heterojunctions helping avoid the photocorrosion through the flow of electrons from Cu_2_O into SnO_2_ [15,17]. NiO possesses a large band gap, rendering it unable to absorb visible light. Lin et al. demonstrated that NiO serves as an effective protective layer for Cu_2_O [18]. Yang et al. focused, among other aspects, on optimizing the thickness of NiO due to its low conductivity, where too thick a layer causes a decrease in the photocurrent value. They also showed that the NiO interlayer indicates a better charge injection from Cu_2_O to the next layer [19]. However, there is no known modification that would enable both stabilizations, achieving high efficiency in CO_2_ reduction and product selectivity. Therefore, in the research work, an attempt was made to synthesize a photocathode using inexpensive readily available materials, as well as a synthesis technique accessible to any laboratory. Two types of properties were utilized for this purpose—the ability for a more efficient charge separation of Al_2_O_3_ [20] and the protective properties of TiO_2_.

Al_2_O_3_ is an insulator with a wide band gap of 7.0 ± 0.1 eV (amorphous form) [21]. Al_2_O_3_ has found application as a passivation layer covering Si in solar cells [22]. Amorphous Al_2_O_3_ (a-Al_2_O_3_) is known for its properties such as its ability to accumulate electrons and suppress surface charge recombination due to the presence of electron acceptor centers (Al^3+^) [23,24].

TiO_2_ is an n-type semiconductor with a wide band gap (3.2 eV) absorbing light from the UV range [25,26]. Titanium (IV) oxide is cheap, non-toxic, easily available, chemically inert, and demonstrates high photostability and resistance to photocorrosion. Unfortunately, its main disadvantage is the rapid recombination of hole–electron pairs [27,28,29,30,31]. The titanium oxide that was used is commercially available P-25, consisting of two polymorphic varieties—anatase and rutile. Mixing these forms in the right proportion (15% anatase, 85% rutile) results in optimal photocatalytic performance and reduced charge recombination through the formation of energy wells, introducing a lower energy level of rutile [32]. One of the most popular techniques for depositing various types of metal oxides is the atomic layer deposition (ALD) method [33,34]. The disadvantage of this technique is the requirement for specialized and expensive equipment, which is not standard in every laboratory.

This work proposes the chemical synthesis of amorphous Al_2_O_3_ and a simple and inexpensive method of depositing Al_2_O_3_ and TiO_2_ layers, available to any chemical laboratory, which is evaporation. It was found that the introduction of Al_2_O_3_/TiO_2_ layers resulted in a significant increase in the photocurrent and an improvement in the stability of the photoelectrode. The chosen electrolyte is 0.5 M NaCl (artificial seawater) due to its abundance. Additionally, it serves to demonstrate the corrosion protection enhancement achieved by the deposited Al_2_O_3_/TiO_2_ layer [35,36]. This allowed for the selective and efficient reduction of CO_2_ to CH_3_OH.

Seawater, as a reaction medium, presents unique challenges due to its high salinity, chloride ion concentration, and presence of various impurities, which can accelerate the photocorrosion of sensitive materials like Cu_2_O. However, experiments have demonstrated that applying protective layers such as Al_2_O_3_ and TiO_2_ can significantly improve the stability of Cu_2_O in 0.5 M NaCl, a corrosive environment mimicking artificial seawater. These coatings act as physical and chemical barriers, preventing the direct exposure of Cu_2_O to aggressive chloride ions and reducing photocorrosion rates. In the literature, one can find studies where the authors focus on the use of seawater as an electrolyte for water and CO_2_ reduction. S. Bai et al. conducted studies in which they demonstrated that ions present in seawater, such as Na^+^, K^+^, Ca^2+^, Mg^2+^, Cl^−^, and Br^−^, influence the process of CO_2_ reduction. The Na^+^_K^+^ mixture in seawater provides a workable buffering capacity that helps regulate the local pH during the CO_2_ reduction reaction. This buffering effect is crucial for maintaining optimal conditions for the reaction, as pH can significantly impact the efficiency and selectivity of CO_2_ reduction products. The interaction of specific anions, such as Cl^−^ and Br^−^, with the copper electrode can lead to microstructural changes that enhance the electrochemical performance. These anions can promote the formation of active sites on the electrode, facilitating the reduction of CO_2_ to desired products. While seawater has many advantages, the presence of ions like Ca^2+^ and Mg^2+^ can lead to precipitation (e.g., calcium carbonate), which can block active sites on the electrode and hinder the CO_2_ reduction process. Therefore, managing these ions is essential for maintaining high performance [37]. Other studies have shown that seawater contains dissolved salts, mainly sodium chloride, which can act as hole scavengers. This prevents the recombination of electron–hole pairs generated during the photocatalytic process, thereby improving the overall efficiency of the reaction. The presence of seawater has been shown to significantly increase the production of formic acid (HCOOH) compared to distilled water. Specifically, the production can be up to 15 times higher in seawater. This enhancement is attributed to the beneficial interactions between the photocatalyst and the components in seawater, which facilitate the reduction of CO_2_. The species present in seawater contribute to improved charge transfer efficiency within the photocatalytic system. This is crucial for the effective conversion of CO_2_ into valuable products like HCOOH and hydrogen (H_2_) [38].

## 2. Materials and Methods

### 2.1. Deposition of Copper (I) Oxide

Fluorine-doped tin oxide (FTO) conductive glass (resistivity, ~8 Ω/sq Sigma-Aldrich, St. Louis, MO, USA) was cleaned by sonication in deionized water with soap, water, ethanol, and water again. Each step took 15 min. After all steps, the electrodes were air dried. Copper (I) oxide was deposited on FTO conductive glass. The technique used was chronoamperometry and the deposition was carried out in a three-electrode system. The electrode substrate was used as the working electrode, a Pt wire as the counter electrode, and a mercury sulfate electrode (MSE, Hg/Hg_2_SO_4_) as the reference electrode. The solution was prepared by dissolving 2.996 g copper sulfate pentahydrate (CuSO_4_-5H_2_O. 98%. Sigma-Aldrich, St. Louis, MO, USA) and 6.7 mL lactic acid (CH_3_CH(OH)CO_2_H. MW: 90.0S g/mol, Sigma-Aldrich, St. Louis, MO, USA) in 23.3 mL Millipore water. The solution was adjusted to pH 9 by adding sodium hydroxide (NaOH. 98.8% POCH, Gliwice, Poland). The potential of the electrode was −0.66 V vs. MSE. The cell containing the working electrode was kept in the bath at 60 °C for 30 min.

### 2.2. Synthesis of Al_2_O_3_

The first step in the synthesis of Al_2_O_3_ was a reaction between aluminum chloride and sodium hydroxide. This was performed using 8.53 g of AlCl_3_ (AlCl_3_ ·6H_2_O. 99% Sigma Aldrich, St. Louis, MO, USA) and 7.69 g of sodium hydroxide (NaOH. 98.8% POCH, Gliwice, Poland) diluted in 100 mL of Millipore water. The sodium hydroxide solution was slowly added to the aluminum chloride, which was cooled in a water bath. After the precipitation of aluminum hydroxide, the precipitate was thoroughly rinsed with Millipore water and later with ethanol. The aluminum hydroxide was then placed in an oven at 550 °C for 3 h to form Al_2_O_3_. Then, 5 g of aluminum oxide was weighed and dissolved in 10 mL of DMF. The vessel containing the solution was sonicated for 30 min.

### 2.3. Preparation of TiO_2_

Commercially available TiO_2_ (P-25) (99.9%. Degussa, Essen, Germany) was dispersed in DMF and sonicated for 30 min to ensure uniform suspension. This TiO_2_ layer serves as an additional protective layer for the Cu_2_O photocathode.

### 2.4. Fabrication of FTO/Cu_2_O/Al_2_O_3_/TiO_2_ Electrodes

The Cu_2_O/Al_2_O_3_/TiO_2_ electrodes were fabricated by spraying pre-prepared suspensions of Al_2_O_3_ and TiO_2_ (dispersed in DMF) onto the Cu_2_O surface using argon gas as the carrier. After deposition, each layer was heated on a hot plate at 150 °C to remove any residual solvent, resulting in a stable layered photocathode structure.

### 2.5. Characterization Methods

An SEM (scanning electron microscopy) analysis of the electrode morphology was conducted to confirm layer uniformity and surface structure, which was carried out using a Carl Zeiss Sigma HV workstation (GmbH, Oberkochen, Germany). The microscope was equipped with a Gemini electron column with an energy-selective backscattered detector and energy-dispersive X-ray spectrometer with Bruker Quantax XFlash 6|10 detectors (GmbH, Karlsruhe, Germany).

UV-Vis spectroscopy was performed using a Jasco V-650 spectrophotometer equipped with a 60 mm integrating sphere (Jasco, Easton, MD, USA). To obtain the spectra, we used diffuse reflectance spectroscopy (DRS) (JASCO Corporation, Hachioji, Tokyo).

The X-ray photoelectron spectra (XPS) were recorded with the use of a PHI 5000 Versa Probe spectrometer (ULVAC-PHI, Chigasaki, Japan). Monochromatic X-rays with the energy hν = 1486.6 eV (AlK_α_) and a power of 23 W were used as the excitation source. Both wide and high-resolution XPS were recorded with the pass energies of 117.4 and 23.5 eV, respectively. Shirley’s background cutoff function was used to determine the intensity of the individual XPS signals. The spectra recorded in a narrow range of binding energies were fitted using the asymmetric Gauss/Lorentz function in CasaXPS software version 2.3. The measured binding energies for all registered elements were corrected in relation to the C 1s carbon peak at 284.8 eV.

Powder X-ray diffraction (XRD) measurements were performed on the X’Pert PRO MPD diffractometer (Malvern Panalytical B.V., Almelo, The Netherlands). The sample was recorded with a step size of 0.026° 2θ using a specimen on a plate, and CoKα radiation was filtered through an iron (Fe) filter with power parameters set at 30 mA and 40 kV. Radiation detection employed a fast linear PIXcel detector (Malvern Panalytival, Eindhoven, The Netherlands).

An analysis of the products was conducted using Tracera GC system GC-2010 Plus (Shimadzu Corporation, Kyoto, Japan) equipped with a mass spectrometer GCMS-QP2020 and BID detector (Shimadzu Corporation, Kyoto, Japan).

### 2.6. Photoelectrochemical (PEC) Measurements

All photoelectrochemical measurements were carried out in a two-compartment Teflon cell with a quartz window (0.5 cm^2^) filled with 0.5 M NaCl (artificial seawater); the electrolyte was purged with argon and CO_2_, respectively, for 30 min before the experiments. A three-electrode system was used, with Ag/AgCl (KCl saturated) as the reference electrode, platinum foil as the counter electrode, and Cu_2_O as the working electrode. The working electrode was illuminated by a solar simulator (Oriel Lamp 150W, Newport, Irvine, CA, USA) equipped with a solar filter. Illumination output was 100 mW/cm^2^. A potentiostat (CH Instruments, Austin, TX, USA, CHI660D) was used to control the potential during PEC measurements. To examine the photoresponse, cyclic voltammetry was used, and the range of applied potential was from 0 V to −0.8 V vs. Ag/AgCl (scan rate 10 mV). I-t measurement was performed by applying −0.5 V vs. Ag/AgCl.

Impedance spectra of synthesized electrodes were recorded at −0.5 V in the 0.05 Hz to 1 MHz frequency range using a sinusoidal excitation signal (5 mV amplitude). Mott–Schottky plots were obtained at one frequency (1 kHz) between E = 0 V to −1 V vs. Ag/AgCl potentials.

## 3. Results and Discussion

**Figure 1:**
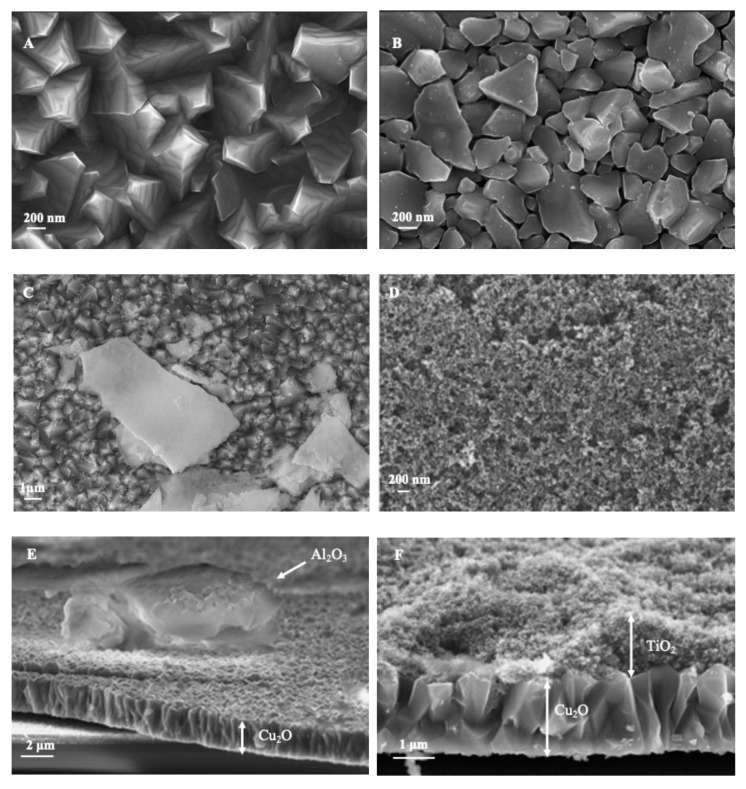
SEM images illustrating (**A**) pristine Cu_2_O, (**B**) pristine Cu_2_O after illumination, (**C**) Cu_2_O/Al_2_O_3_, (**D**) Cu_2_O/Al_2_O_3_/TiO_2_, (**E**) cross-section of Cu_2_O/Al_2_O_3_/TiO_2_, (**F**) cross-section of Cu_2_O/Al_2_O_3_/TiO_2_.

The main component of the photoelectrochemical cell is a Cu_2_O-based semiconductor material. Due to its instability, Cu_2_O was modified with different layers. SEM images of the prepared electrodes are shown in Figure 1.

The electrochemical deposition of Cu_2_O onto the FTO substrate yielded uniform coverage by Cu_2_O crystallites (Figure 1A,B), which exhibit a columnar pyramidal morphology. The growth of these crystallites is highly regular, and the cross-sectional analysis reveals minimal spacing between them, contributing to reduced surface roughness. The sputtered Al_2_O_3_ layer (Figure 1C) forms a non-uniform coating resembling irregular platelet, creating a thin oxide layer on top of the Cu_2_O, as shown in the inset. This Al_2_O_3_ layer is expected to improve the charge transport properties of Cu_2_O without significantly enhancing its stability, as reported in previous studies [39,40].

Complete and uniform coverage of Cu_2_O and Al_2_O_3_ layers was achieved with the addition of TiO_2_ (Figure 1D), providing enhanced structural integrity. The layer comprises densely packed TiO_2_ nanoparticles with small interconnected pores, which stabilize the Cu_2_O and mitigate photocorrosion effects [41,42,43]. In the cross-sectional SEM image of Cu_2_O/Al_2_O_3_ (Figure 1E), a continuous 1.8 μm thick Cu_2_O layer is observed, along with a minimal Al_2_O_3_ coating across the electrode surface. Furthermore, a continuous TiO_2_ layer approximately 1 μm thick covers the Cu_2_O/Al_2_O_3_ electrode, as shown in Figure 1F, providing electrolyte permeability. Corresponding EDS maps and spectra for each layer, along with detailed descriptions, are provided in the Supporting Information in Figure S1 to further confirm the elemental composition and uniformity of the coatings.

The X-ray diffraction (XRD) patterns of the Al_2_O_3_ powder and the Cu_2_O/Al_2_O_3_/TiO_2_ electrode, both before and after illumination (see Figure S2), display characteristic structural peaks at 2θ values of 42°, 50°, and 72°, corresponding to the Cu_2_O planes, and at 2θ values of 29° and 32° for TiO_2_. Notably, post-photoelectrochemical treatment reveals reduced peak intensities, suggesting partial degradation and depletion of the active layer. Al_2_O_3_ remains amorphous, as indicated by the absence of α-, β-, or γ-polymorph peaks. SEM and XRD analyses confirm the partial reduction of Cu_2_O to metallic copper, which may result from restructuring or delamination processes during operation.

One possible explanation for these observations is a restructuring of the surface layer, potentially accompanied by recrystallization. Partial degradation and delamination of the thin film may also contribute. Scanning electron microscopy (SEM) analysis confirms the presence of TiO_2_ and Al_2_O_3_ overlayers following the reduction process; however, a clear reduction of some Cu_2_O to metallic copper is observed.

**Figure 2:**
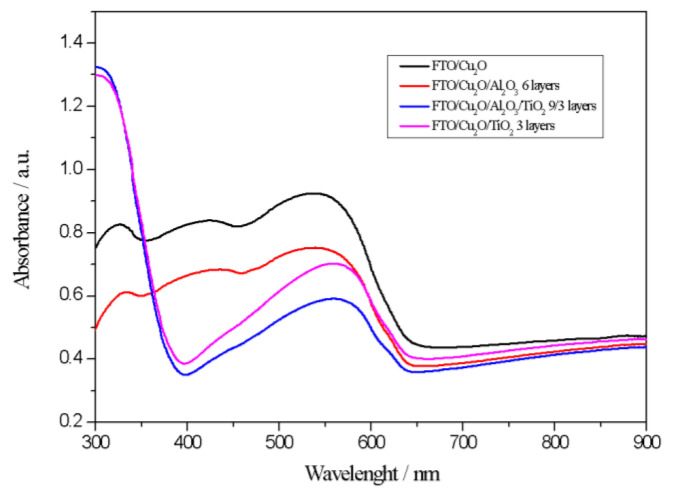
Absorbance spectra of UV-Vis of Cu_2_O-based photocathodes.

Figure 2 presents the absorbance spectra as a function of wavelength for the FTO/Cu_2_O/Al_2_O_3_/TiO_2_ photocathode and comparative systems. The black curve corresponds to the baseline FTO/Cu_2_O photocathode, which displays a characteristic absorption band centered around 550 nm [44], consistent with the known visible light absorption properties of Cu_2_O [42]. The red curve represents the FTO/Cu_2_O/Al_2_O_3_ photocathode with six layers, where a slight decrease in intensity is noted compared to the FTO/Cu_2_O sample, likely attributed to the presence of Al_2_O_3_. Nevertheless, the dominant feature remains as the Cu_2_O absorption band.

For the other two systems, a subtle shift towards longer wavelengths is observed, which can be ascribed to the incorporation of TiO_2_. Titanium (IV) oxide exhibits light absorption primarily in the ultraviolet range, as indicated by the profiles of the absorbance curves [45]. Additionally, due to the wide band gap of Al_2_O_3_, this material absorbs light in the far ultraviolet range, which is not represented in the spectra shown above [46].

**Figure 3:**
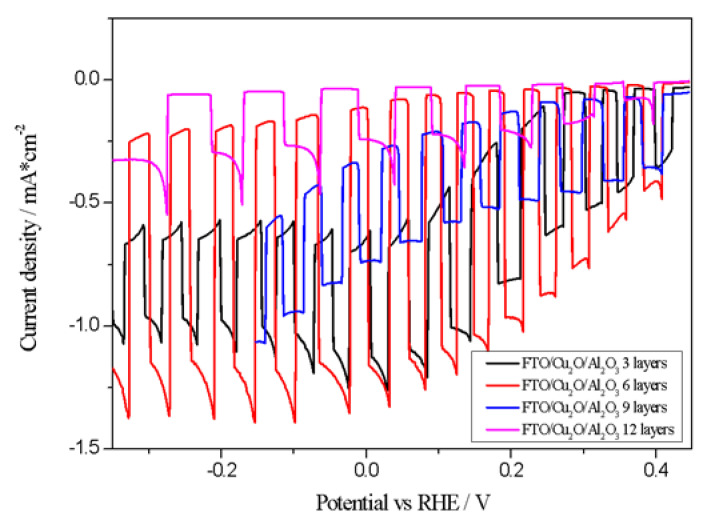
Photocurrent density as a function of applied potential (vs. RHE) in 0.5 M NaCl-saturated CO_2_ solution.

To determine the most effective thickness of Al_2_O_3_, PEC experiments were conducted with electrodes prepared using varying Al_2_O_3_ layer numbers (3, 6, 9, and 12), as illustrated in Figure 3. 

**Figure 4:**
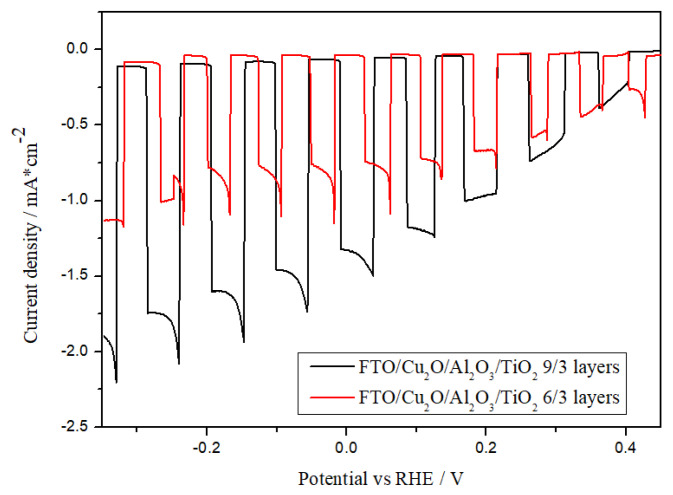
Photocurrent density as a function of potential (vs. RHE) in 0.5 M NaCl-saturated CO_2_ solution.

As shown in Figure 4, an insufficient Al_2_O_3_ layer (three layers) yields limited CO_2_ reduction activity. In contrast, the Cu_2_O/Al_2_O_3_ photocathode with six layers achieved the highest photocurrent density (−0.1 mA/cm^2^ at −0.2 V vs. RHE), which is attributed to increased charge carriers and enhanced transport. Higher layer counts, such as nine or twelve, resulted in reduced photocurrents, likely due to hindered electron transport and increased recombination events (observed as spiking behavior). The introduction of a TiO_2_ overlayer further improved electrode stability and increased photocurrent density to −1.4 mA/cm^2^ at −0.2 V vs. RHE, as demonstrated in Figure 4.

**Figure 5:**
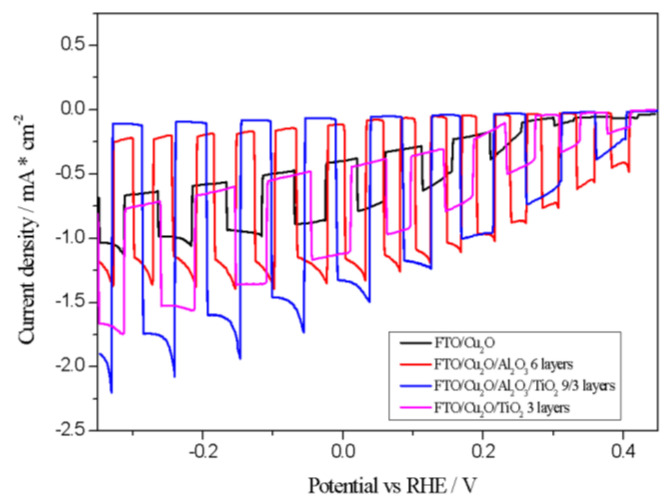
Photocurrent density versus potential (vs. RHE) in 0.5 M NaCl-saturated CO_2_ solution.

Significant photocurrent differences were observed between the six-layer and nine-layer Al_2_O_3_ configurations. Therefore, further experiments were focused on the Cu_2_O/Al_2_O_3_/TiO_2_ electrode with a nine-/three-layer distribution. As shown in Figure 5, the addition of Al_2_O_3_ and TiO_2_ layers reduces the dark current, which refers to the electrical current that flows through the system in the absence of light illumination. Its origin primarily lies in two factors. First, it can result from purely electrochemical reactions occurring at the electrode surface, such as the reduction of CO_2_ or water splitting reactions, including the hydrogen evolution reaction. Second, dark current may arise from the corrosion or degradation of the electrode material itself. This is particularly relevant for materials like Cu_2_O, which are susceptible to corrosion. Al_2_O_3_ and TiO_2_ layers suppress electrochemical degradation and enhance photocurrent generation efficiency.

For a more comprehensive stability assessment, a one-hour I-t measurement was conducted at a constant potential of 0 V vs. RHE. The unstable nature of Cu_2_O in aqueous electrolytes is visually confirmed by changes in crystal structure over time, as seen in SEM images.

**Figure 6:**
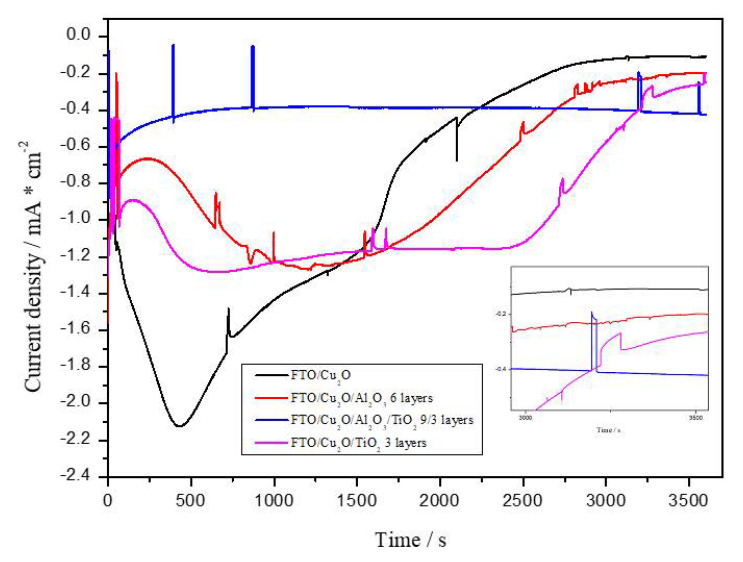
Photocurrent density versus time (I-t) curves in 0.5 M NaCl-saturated CO_2_ solution.

Figure 6 shows that the most rapid and fastest self-reduction of the Cu_2_O surface occurs when it is not modified, resulting in practically no photoresponse after an hour of measurement at a constant potential. The introduction of an Al_2_O_3_ layer does not hinder this process, although it does prolong it over time. Similarly, TiO_2_ acts independently and performs better as a protector of Cu_2_O, further limiting the semiconductor’s reduction over time. In the case of Al_2_O_3_/TiO_2_ layers, this process is most significantly inhibited and the dark current values for the tested electrode are the lowest, indicating that the self-oxidation and reduction processes of Cu_2_O are curbed. There is also a noticeable increase in photocurrents generated on the FTO/Cu_2_O/Al_2_O_3_/TiO_2_ photocathode, which are three times higher than those on the unmodified Cu_2_O photocathode, with values of 0.18 mA·cm^−2^ and 0.06 mA·cm^−2^, respectively. As reported in many works, the reason for this may be the facilitated interfacial transport of charges and the improved separation of charges by introducing the Al_2_O_3_/TiO_2_ system.

The introduction of Al_2_O_3_/TiO_2_ layers results in a faster photoelectrochemical response, which can be recognized by the shape of the blue curve and its symmetric rectangular shape. The characteristic shapes of the curves for Cu_2_O/Al_2_O_3_ and Cu_2_O/TiO_2_ electrodes may be caused by a rapid change in the degree of oxidation from Cu^+^ to Cu^2+^, which is associated with the degradation of the electrode and a reduction in its efficiency. This may be due to the accumulation of excess charges, which are more susceptible to recombination of the resulting hole–electron pairs or oxidation of the Cu_2_O electrode surface itself.

Figure 6 illustrates the stability and photoelectrochemical performance of various Cu_2_O-based electrodes, focusing on the effect of Al_2_O_3_ and TiO_2_ layers on the self-reduction and photocurrent generation of Cu_2_O. The unmodified Cu_2_O surface undergoes the fastest self-reduction, as evidenced by the significant decay in its photoresponse, resulting in negligible photocurrent after one hour of measurement at constant potential. This behavior demonstrates the inherent instability of the unprotected Cu_2_O electrode under operational conditions.

The introduction of an Al_2_O_3_ layer partially mitigates this issue by prolonging the reduction process, although it does not entirely prevent it. TiO_2_, on the other hand, acts as a more effective stabilizer for the Cu_2_O surface, limiting the extent of reduction over time and offering better protection. However, the most substantial improvement is observed when both Al_2_O_3_ and TiO_2_ layers are applied together. This dual-layer system significantly suppresses the self-reduction and self-oxidation processes of Cu_2_O, as indicated by the low dark current values measured for the FTO/Cu_2_O/Al_2_O_3_/TiO_2_ electrode.

Additionally, the photocurrents generated by the FTO/Cu_2_O/Al_2_O_3_/TiO_2_ photocathode are three times higher than those of the unmodified Cu_2_O electrode, with values of 0.18 mA/cm^2^ and 0.06 mA/cm^2^, respectively. This increase in photocurrent suggests enhanced charge transport and separation at the interface due to the introduction of the Al_2_O_3_/TiO_2_ system, as also reported in previous studies [47,48,49].

**Figure 7:**
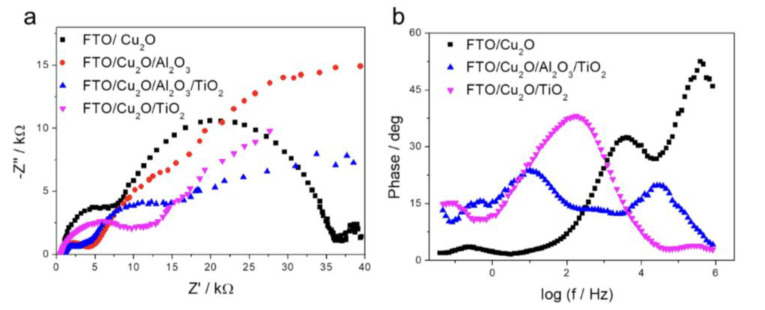
(**a**) Nyquist plots from EIS of bare Cu_2_O, Cu_2_O/Al_2_O_3_, Cu_2_O/TiO_2_, and Cu_2_O/Al_2_O_3_/TiO_2_ electrodes; (**b**) Bode plot from EIS of bare Cu_2_O, Cu_2_O/TiO_2_, and Cu_2_O/Al_2_O_3_/TiO_2_ electrodes.

An EIS analysis of photocathodes is presented in Figure 7. In the Nyquist plots for all systems, two distinct semicircles are observed. The first semicircle is interpreted as representing interfacial processes at the electrode–electrolyte boundary. Specifically, in the context of CO_2_ photoreduction, this first semicircle is associated with charge transfer processes occurring at the electrode surface, where electrons transfer between the electrode and CO_2_ molecules. The second semicircle, present across all systems, reflects mass transport processes. A detailed examination of the first semicircle reveals that charge accumulation and charge transfer resistance are both highest for the unmodified Cu_2_O system, as well as for the system modified with TiO_2_ alone. Notably, the introduction of an Al_2_O_3_ layer consistently and significantly reduces the surface charge accumulation (indicated by a smaller diameter of the first semicircle, which corresponds to the capacitance of the generated double-layer capacitor) and decreases charge transfer resistance. The combined Al_2_O_3_/TiO_2_ layer further enhances product distribution by reducing the diameter of the second semicircle, indicative of improved mass transport characteristics [50].

The recorded Bode curves in Figure 7b (black curve for Cu_2_O) reveal two primary electrochemical processes, which we interpret as CO_2_ reduction and Cu_2_O electrode photocorrosion. The first peak is associated with the CO_2_ reduction process, occurring at lower frequencies and displaying notable kinetics. The second more intense and rapid peak is attributed to photocorrosion of the Cu_2_O photocathode. Coating the electrode with a TiO_2_ film significantly decreases the photocorrosion peak amplitude and shifts the CO_2_ reduction kinetics by an order of magnitude (pink curve). Conversely, the Al_2_O_3_/TiO_2_ system further slows CO_2_ reduction kinetics, though a slight increase in Cu_2_O photocathode photoreduction is observed.

These findings suggest that CO_2_ reduction on a pure Cu_2_O electrode proceeds rapidly, likely favoring water reduction with partial CO formation. However, modification with TiO_2_ and Al_2_O_3_ films markedly slows the CO_2_ reduction process, which may allow for the formation of more complex products such as CH_3_OH and inhibit competitive water reduction reactions. A key distinction between the Al_2_O_3_/TiO_2_ and TiO_2_-only systems is seen in the full width at half maximum (FWHM) of the peaks: the pure TiO_2_ system exhibits a broader peak, indicative of multiple heterogeneous processes, while the Al_2_O_3_/TiO_2_ system displays a narrower FWHM, suggesting a more selective process. The suitable equivalent circuit diagrams of examined electrodes are in Si in Figure S3.

The Al_2_O_3_ layer acts as a passivation layer that reduces surface charge accumulation and minimizes recombination losses at the Cu_2_O surface. This is evidenced by the reduced diameter of the first semicircle in the Nyquist plots. Al_2_O_3_ likely forms a physical barrier that moderates the interaction between the Cu_2_O surface and the electrolyte, leading to lower charge transfer resistance at the electrode/electrolyte interface. This passivation effect also suppresses photocorrosion, as observed in the Bode plot, by protecting the Cu_2_O surface from oxidative degradation. The TiO_2_ layer enhances the separation and mobility of photogenerated charge carriers. It forms a heterojunction with the Cu_2_O layer, creating an electric field that facilitates electron transfer from Cu_2_O to TiO_2_. This is supported by the observed improvement in CO_2_ reduction kinetics in the Bode plot, where the TiO_2_ layer shifts the reduction peak and reduces the photocorrosion signal. The presence of TiO_2_ also contributes to improved stability by scavenging photogenerated holes, further reducing degradation of the Cu_2_O layer. The combination of Al_2_O_3_ and TiO_2_ layers results in a cooperative effect that enhances both charge transfer and mass transport processes. The Al_2_O_3_ layer ensures lower charge accumulation, while the TiO_2_ layer provides a pathway for efficient electron transfer. This synergy is evident in the Nyquist plots, where the first semicircle (charge transfer) and the second semicircle (mass transport) are both reduced in the trilayered system compared to single-layer systems. The improved mass transport characteristics observed for the trilayered system could be attributed to the optimized surface properties and reduced resistance at the interface.

**Figure 8:**
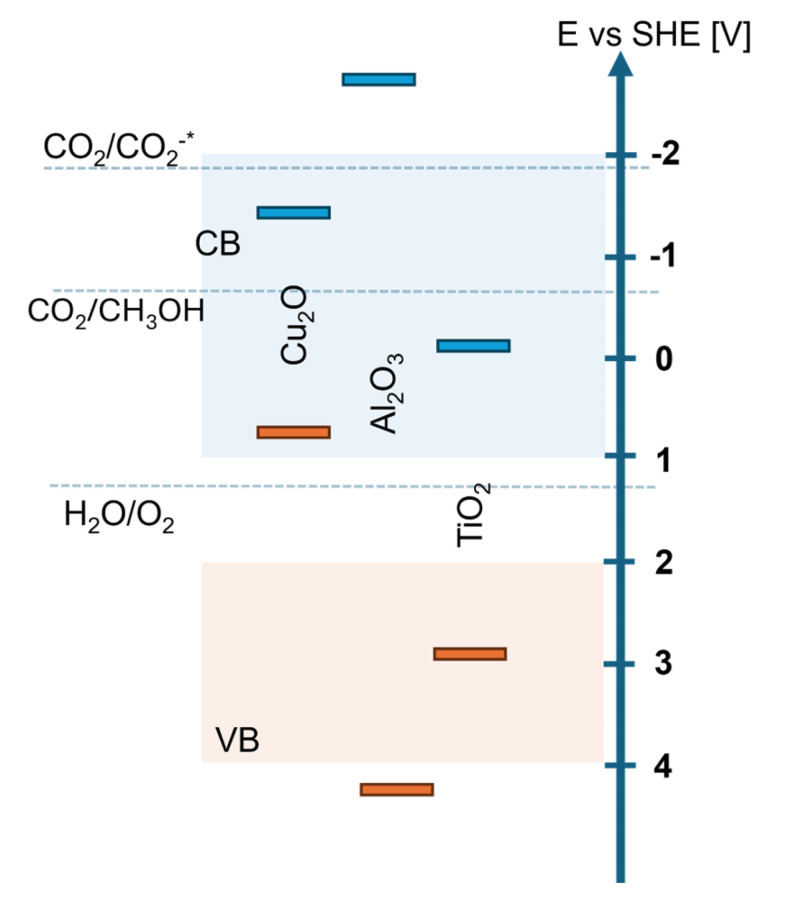
Schematic representation of the arrangement of the Cu_2_O, Al_2_O_3_, and TiO_2_ layers, along with their respective energy levels.

We have included a schematic representation of the arrangement of the Cu_2_O, Al_2_O_3_, and TiO_2_ layers, along with their respective energy levels (valence band (VB) and conduction band (CB)) and potentials. The diagram in Figure 8 clearly illustrates the alignment of the materials’ energy bands, highlighting their relative positions and the electron/hole transfer processes. This additional figure provides a comprehensive understanding of the system’s structure and the energy potentials of each layer.

The conduction band position of Cu_2_O allows for the efficient transport of electron carriers to the TiO_2_ layer, facilitating the desired charge transfer processes within the system. Additionally, the Al_2_O_3_ layer plays a crucial role in stabilizing the entire structure due to its excellent chemical resistance. This layer acts as a protective barrier, preventing potential degradation of the active materials and ensuring the long-term durability and functionality of the system. Moreover, Al_2_O_3_ contributes to maintaining the structural integrity of the device, especially under challenging environmental conditions, thereby enhancing its overall reliability and performance. This synergistic interaction between the Cu_2_O, Al_2_O_3_, and TiO_2_ layers ensures an optimal combination of charge transfer efficiency, chemical stability, and durability.

**Figure 9:**
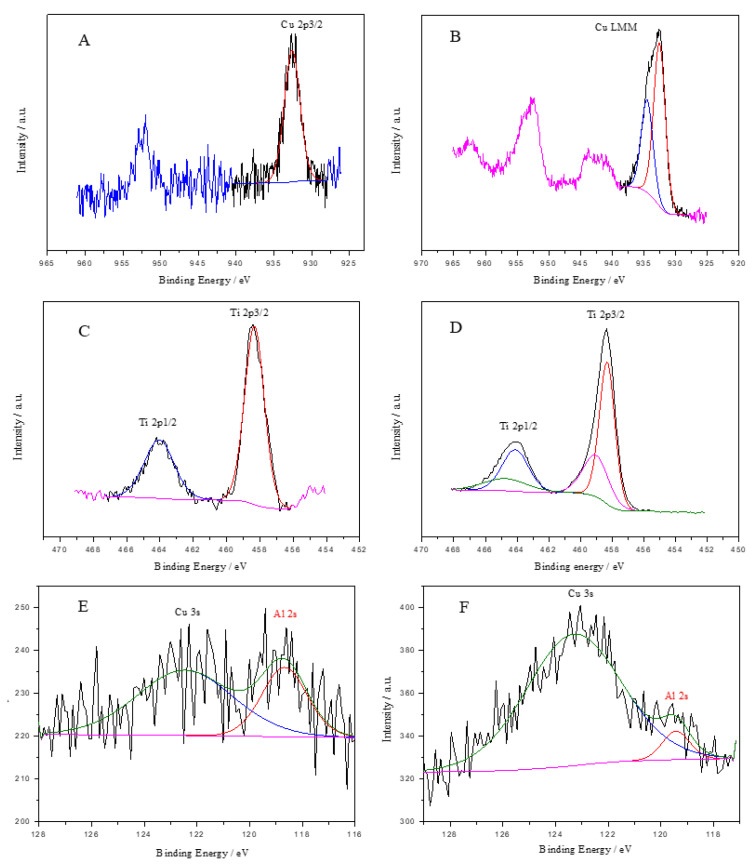
High-resolution XPS of (**A**) Cu 2p before experiment, red line (**B**) after experiment, red and blue line, (**C**) Ti 2p before experiment red and blue line (**D**) after experiment, red, blue and pink line, and Al 2s (**E**) before experiment, red line and (**F**) after experiment, red line were recorded on the Cu_2_O/Al_2_O_3_/TiO_2_ surface before and after illumination.

To fully understand the chemical form of the manufactured material surfaces before and after illumination, XPS measurements were carried out. The maximum energy of the Cu2p_3/2_ peak is 932.6 eV, and its position and shape correspond to the presence of copper (I) oxide in both cases (Figure 9A,B). However, some differences can be observed after irradiation. There is an additional copper signal that can be attributed to the presence of Cu^2+^ (Figure 9B). The presence of such an oxidized copper form is an undesirable but well-known phenomenon in this type of investigation [51]. The main peak position located at 458.3 eV and 464.1 eV corresponds to the binding energy of Ti 2p_3/2_ and 2p_1/2_, respectively, which is typical for Ti^4+^ (Figure 9C,D). It should be noted that the reference position of titanium (IV) oxide is usually 458.8 eV [52]. This distinct shift of about 0.5 eV may be caused by the formation of a Ti-O-Al heterojunction between Al_2_O_3_ and TiO_2_ oxides [53]. This behavior may also be affected by the presence of copper on the heterojunction surface [54]. Also, in the case of titanium after irradiation, there has been a change in the chemical state of this element; in addition to the peak characteristic of TiO_2_, an asymmetric peak located at 459.0 eV is visible, which indicates a change in the electron structure of Ti due to the presence of a heterojunction (Figure 9D) [53,54,55,56].

Determining the chemical state of aluminum before and after the reaction using light was difficult because of the overlapping of the signals of Cu3s with Al2s and Cu3p with Al2p. After the deconvolution procedure, the position of the Al2s peak was assigned at 118.7 eV and 119.4 eV in the presence of Al_2_O_3_ oxide (Figure 9E,F). It was also noticed that its amount decreased significantly after illumination compared to other metals, which may indicate its dropout from the photocathode surface. A detailed description of the spectral analysis of oxygen, including the interpretation of its speciation in the ‘before’ and ‘after’ samples, is provided in the Supplementary Information.

Finally, an attempt was made to identify the products of CO_2_ reduction on the FTO/Cu_2_O/Al_2_O_3_/TiO_2_ photocathode using gas chromatography. The expected product of PEC CO_2_ reduction on the resulting electrode is methanol due to the outer layer of TiO_2_ also acting as a catalyst for the reduction of CO_2_ to CH_3_OH [57,58]. GCMS analysis confirmed the production of methanol with 69% Faradaic efficiency, which was calculated by the following equation:

(1) $$FE=\frac{ze^{-}\times n_{CH3OH}\times F}{Q}\times 100\%$$

(2) $${{CO}_{2}+6H^{+}+{6e}^{-}\to \;CH}_{3}OH+H_{2}O$$

*ze*^−^ is the number of electrons needed for CO_2_ reduction into CH_3_OH, *n_CH3OH_* is the molar number of generated methanol, *F* is the Faraday constant, and *Q* is the charge that has passed during the experiment.

Ibadillah A. Digdaya et al. conducted studies and demonstrated that the efficiency of CO_2_ reduction (CO2R) in the described system can reach a total Faradaic efficiency (FE) of up to 95% for the conversion of CO_2_ to CO using a silver (Ag) electrocatalyst. Additionally, when using a copper (Cu) electrocatalyst, the system can achieve a total Faradaic efficiency of up to 73% for converting CO_2_ into various fuels (CH_3_OH, C_2_H_4_, CH_4_, HCOOH) [59].

Nakata et al. reported that the efficiency of CO_2_ reduction using the boron-doped diamond electrode is reported to have a Faradaic efficiency of 74% to produce formaldehyde. As for the electrolyte, they utilized seawater, which acts as a source of both electrons and protons for the reduction process [60].

Kim et al. observed Faradaic efficiencies of 62% for formic acid, 85% for formaldehyde, 8% for methanol, and 6% for ethanol at different bias potentials in the 0.5 M NaCl electrolyte. The electrode system utilized in the CO_2_ reduction reaction consists of a (040) facet engineered BiVO_4_ (040-BVO) as the photoanode (working electrode) and copper (Cu) foil as the cathode (counter electrode). Copper is noted as the only active metallic electrocatalyst that produces formic acid, formaldehyde, and alcohols as major products via the CO_2_ reduction reaction. The electrolyte used in the experiments was a 0.5 M NaCl solution. This electrolyte was chosen due to its conductivity and the presence of chloride ions, which help reduce double-layer capacitance. The study highlights the efficiency of CO_2_ reduction using a specific electrode setup and electrolyte, showcasing the potential for producing valuable chemical fuels through artificial photosynthesis [61].

The 69% Faradaic efficiency reported in our study is consistent with efficiencies achieved in other CO_2_ reduction systems using different electrocatalysts but the same electrolyte (artificial sweater, oceanwater). This result confirms the reliability of the Cu_2_O/Al_2_O_3_/TiO_2_ electrode and highlights its potential for practical applications in CO_2_ reduction using artificial seawater.

To compare the products generated across all electrodes, we experimented using a second gas chromatography system. However, this system displayed substantially higher sensitivity to hydrogen, which influenced our results, as presented in Figure S4. The data reveal a pronounced decrease in hydrogen production on modified electrodes, alongside significantly improved selectivity on the Al_2_O_3_-coated electrode, as evidenced by the markedly lower levels of CO. This enhanced selectivity highlights the effectiveness of the Al_2_O_3_ modification in suppressing unwanted by-products, suggesting its potential for applications where hydrogen purity and CO minimization are critical.

## 4. Conclusions

In summary, the FTO/Cu_2_O/Al_2_O_3_/TiO_2_ photocathode was successfully obtained, which is characterized by a simple and easily accessible synthesis technique that does not require highly sophisticated and expensive techniques such as those commonly used in Al_2_O_3_ ALD. The introduction of additional Al_2_O_3_ and TiO_2_ layers improved the electrode’s stability, increased its photoelectrochemical activity, and led to effective charge separation and electron lifetime extension, resulting in an increase in photocurrent density from 0.5 mA/cm^−2^ for FTO/Cu_2_O to 1.4 mA/cm^−2^ for FTO/Cu_2_O/Al_2_O_3_/TiO_2_ at a potential of −0.2 V vs. RHE, which is a 2.8-fold increase in photocurrent density. The middle Al_2_O_3_ layer may support the separation of hole–electron pairs and their further transport to TiO_2_ and then to the electrolyte.

## Data Availability

The original contributions presented in the study are included in the article/Supplementary Materials, further inquiries can be directed to the corresponding author.

## Supplementary Materials

The following supporting information can be downloaded at: https://www.mdpi.com/article/10.3390/ma18030620/s1, Figure S1: EDS analysis of (A) FTO/Cu_2_O/Al_2_O_3_ (B), (C,D) FTO/Cu_2_O/Al_2_O_3_/TiO_2._; Figure S2: XRD patterns of Al_2_O_3_ (blue)—powder and Cu_2_O/Al_2_O_3_/TiO_2_ before (black) and after experiment (red).; Figure S3: Suitable circuit diagram of examined electrodes.; Figure S4: CO_2_ reduction product distribution over Cu_2_O (0.5 M NaCl saturated by CO_2_), Cu_2_O/TiO_2_ (0.5 M NaCl saturated by CO_2_), Cu_2_O/Al_2_O_3_/TiO_2_ (0.5 M NaCl saturated by CO_2_).
