# Supplementary material for: Efficient Photoelectrochemical Reduction of CO2 in Seawater with Cheap and Abundant Cu2O/Al2O3/TiO2 Electrode
Source: Materials (Basel). 2025 Jan 29;18(3):620. doi: 10.3390/ma18030620 (PMC11818597; doi:10.3390/ma18030620)
Supplement: Supplementary file 1 [file materials-18-00620-s001.zip › materials-3382762-supplementary.pdf]

Efficient photoelectrochemical reduction of CO_2 in seawater with cheap and abundant $Cu_2O/Al_2O_3/TiO_2$ electrode

Aleksandra Parzuch[1], Katarzyna Kuder[1] , Kostiantyn Nikiforow[2] , Piotr Wróbel[3] , Grzegorz Kaproń[4] , Krzysztof Bieńkowski[1] , Renata Solarska[1]*

[1]Laboratory of Molecular Research for Solar Energy Innovations, Centre of New Technologies University of Warsaw, Banacha 2c, Warsaw, 02-097, Poland
[2]Institute of Physical Chemistry Polish Academy of Science, Kasprzaka 44/52, 01-224 Warsaw, Poland
[3]Faculty of Physics, University of Warsaw, Warsaw, 02-093, Poland
[4]Faculty of Geology, University of Warsaw, ul. Żwirki i Wigury 93, Warsaw, 02-089, Poland

e-mail: a.parzuch@cent.uw.edu.pl; r.solarska@cent.uw.edu.pl

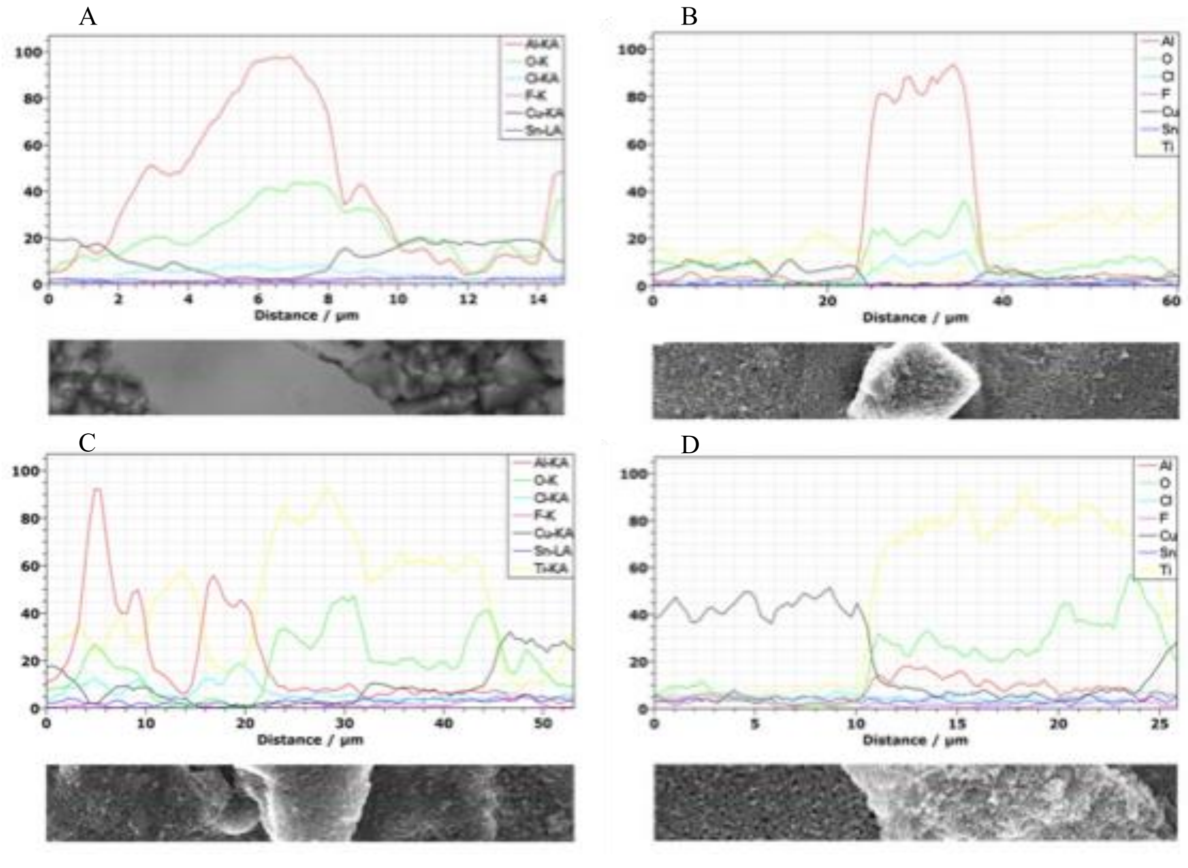

Figure S1: EDS analysis of A) $FTO/Cu_2O/Al_2O_3$ B), C) and D) $FTO/Cu_2O/Al_2O_3/TiO_2$.

The EDS analysis presented in **Figure S1** allows for the identification of individual layers in the system consisting of electrochemically deposited Cu_2O on an FTO substrate, with sputtered Al_2O_3 and TiO_2 layers. The figure shows the EDS profile for an area with a dominant presence of aluminum and oxygen, confirming the presence of Al_2O_3 crystal. These peaks are significantly more pronounced than the signals from other elements (e.g., Cu, Sn, F), which may originate from the underlying layers (Cu_2O or FTO). The SEM image below the graph shows a large crystal consistent with the chemical analysis

results. In **Figure S1 B**, a similar chemical composition profile is visible, dominated by Al and O, again confirming the presence of the Al_2O_3 layer. A low signal for Ti indicates that TiO_2 does not completely cover the underlying Al_2O_3 layer. **Figure S1 C**, distinct signals for Cu and F appear, indicating the presence of the Cu_2O layer deposited on the FTO substrate. The Al and O peaks indicate the covering Al_2O_3 layer, which is visible but does not dominate over the Cu signal. A low Ti signal suggests the presence of a thin TiO_2 layer on the surface, but it is less significant in this area of analysis. **Figure S1 D**, the EDS profile clearly reveals the presence of Ti and O as dominant elements, indicating the TiO_2 layer sputtered over the entire system. Lower signals for Al and Cu indicate the underlying Al_2O_3 and Cu_2O layers, while a low Sn signal originates from the FTO substrate. The SEM image in this section shows a surface where the TiO_2 layer is most prominent.

EDS analysis confirms the structure of the multilayer system consisting of FTO, Cu_2O, Al_2O_3, and TiO_2, with each layer precisely identified through elemental profiles in the respective graphs.

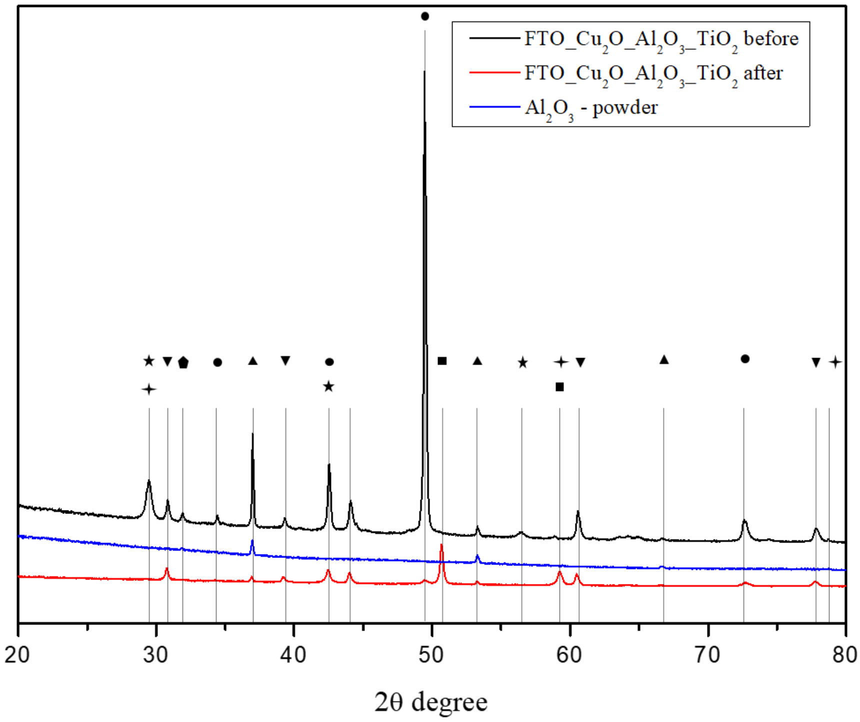

Figure S2: XRD patterns of Al_2O_3 (blue) - powder and $Cu_2O/Al_2O_3/TiO_2$ before (black) and after experiment (red).

The X-ray diffraction (XRD) patterns of the Al_2O_3 powder and the $Cu_2O/Al_2O_3/TiO_2$ electrode, both before and after illumination **Figure S2**, display characteristic structural peaks at 2θ values of 42°, 50°, and 72°, corresponding to the Cu_2O planes, and at 2θ values of 29° and 32° for TiO_2. Notably, post-photoelectrochemical treatment reveals reduced peak intensities, suggesting partial degradation and depletion of the active layer. Al_2O_3 remains amorphous, as indicated by the absence of α-, β-, or γ-polymorph peaks. SEM and XRD analyses confirm partial reduction of Cu_2O to metallic copper, which may result from restructuring or delamination processes during operation. JPCDS ●Cu_2O (00-005-0667)

▼SnO_2 (01-077-0452) ▲NaCl (00-005-0628) ■Cu (00-003-1005)★TiO_2 anatase (01-086-1157) ⬟ TiO_2 rutile (01-075-1750) ✚Al_2O_3 (01-077-2135).

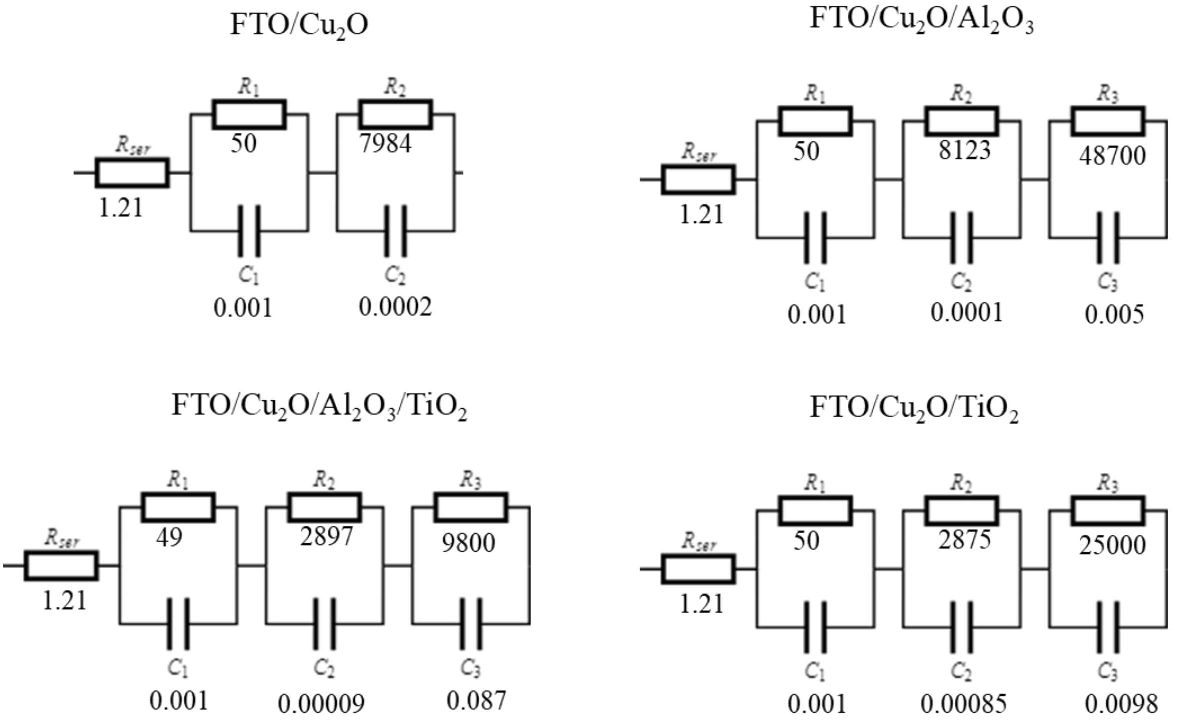

Figure S3: Suitable circuit diagram of examined electrodes.

Equivalent plots for all systems have been added as **Figure S3.** Please note that EIS analysis is inherently ambiguous, and multiple equivalent circuit models can be fitted to the obtained data. The authors have tried to employ the simplest possible models; however, the complex nature of the processes occurring at the electrode, as well as the variety of reactions, including the photodegradation of the semiconductor electrode, must be considered. Therefore, the authors believe that a detailed analysis and precise fitting of equivalent circuits could form the basis for a separate, dedicated publication.

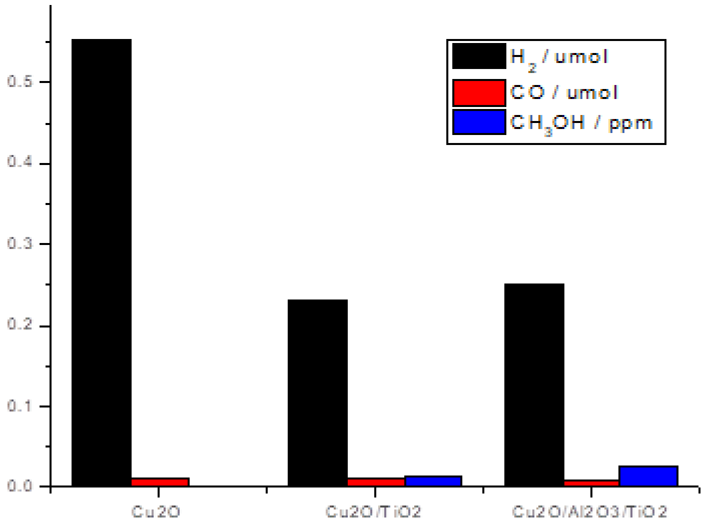

Figure S4: CO_2 reduction product distribution over Cu_2O (0.5M NaCl saturated by CO_2), Cu_2O/TiO_2 (0.5M NaCl saturated by CO_2), $Cu_2O/Al_2O_3/TiO_2$ (0.5M NaCl saturated by CO_2).

To compare the products generated across all electrodes, we conducted an experiment using a second gas chromatography system. However, this system displayed substantially higher sensitivity to hydrogen, which influenced our results, as presented in **Figure S4**. The data reveal a pronounced decrease in hydrogen production on modified electrodes, alongside significantly improved selectivity on the Al_2O_3-coated electrode, as evidenced by the markedly lower levels of CO. This enhanced selectivity highlights the effectiveness of the Al_2O_3 modification in suppressing unwanted by-products, suggesting its potential for applications where hydrogen purity and CO minimization are critical.

The selectivity of the Cu_2O-based samples was evaluated by analyzing the production of H_2, CO, and CH_3OH, as shown in **Figure S4**. Among the tested materials, Cu_2O exhibited the highest H_2 evolution, with minimal CO generation, indicating a strong preference for water splitting rather than Cu_2O reduction.

The Cu_2O/TiO_2 composite showed a reduction in H2 evolution compared to pristine Cu_2O, notably, CH_3OH production appeared for the first time in this system. This result suggests that the addition of TiO_2 plays a crucial role in introducing selectivity toward CO_2 reduction, even if the overall CH_3OH levels remain low.

Notably, the $Cu_2O/Al_2O_3/TiO_2$ system demonstrated the best balance between H2 and carbonaceous product selectivity. While H_2 production is still the dominant process, this sample exhibited significantly higher production of CH_3OH compared to the other materials, indicating improved activity for CO_2 conversion. The CO production remained low across all samples, suggesting that the catalytic systems favor CH_3OH over CO in the CO_2 reduction process.

The reference sample exhibited negligible activity for both H_2 and carbon-containing products, confirming that the observed photo electrocatalytic performance is intrinsic to the Cu_2O-based materials. These results highlight the importance of material composition and the synergistic effects of the

$Cu_2O/Al_2O_3/TiO_2$ layers in enhancing the selectivity and activity for CO_2 reduction.

In our study, the oxygen species in the “before” sample are influenced by a high sodium content, which complicates interpretation due to the presence of intense Na KLL Auger peaks that overlap with the O1s region. Nevertheless, two prominent oxygen peaks are clearly distinguishable. The first peak, located at 529.6 eV, corresponds to lattice oxygen from metal oxides present in the sample. The second peak, at 531.7 eV, may be attributed to C=O groups, as supported by a corresponding peak at 287.2 eV in the C1s spectrum, or to surface hydroxyl groups (OH^-/O_2) as suggested in the referenced paper. The positions of these peaks align with those inferred from the supplemental data in the article, even though specific numerical values were not provided. Unfortunately, due to the interference of Na KLL peaks above 533 eV, we cannot determine whether peaks corresponding to other oxygen species, such as O_2^{2-}/O^-, are present. In the “after” sample, the sodium content is significantly reduced, which eliminates the interference of Na KLL peaks in the O1s region and allows for a clearer interpretation of the spectrum. The O 1s spectrum for this sample was fitted with three peaks. The first, at 529.6 eV, corresponds to lattice oxygen, consistent with the “before” sample. The second peak, at 530.9 eV, is shifted to a lower binding energy compared to the “before” sample, suggesting the presence of hydroxyl groups, adsorbed oxygen, or oxygen vacancies rather than C=O groups. The third peak, at 532.0 eV, is likely attributed to C=O groups. The article referenced by the reviewer suggests that O_2^{2-}/O^- species, with a peak near approximately 533.3 eV (as inferred from Fig. S5), are critical to photocatalytic activity. However, in our study, such a peak is absent in the “after” sample, indicating that O_2^{2-}/O^- species are not present. In the “before” sample, it is not possible to confirm the presence or absence of this peak due to the overlapping Na KLL peaks.
